# Current status of Cancer Nanotheranostics: Emerging strategies for cancer management

**DOI:** 10.7150/ntno.82263

**Published:** 2023-05-01

**Authors:** Vivek P Chavda, Avinash Khadela, Yasha Shah, Humzah Postwala, Pankti Balar, Lalit Vora

**Affiliations:** 1 Department of Pharmaceutics and Pharmaceutical Technology, L.M. College of Pharmacy, Ahmedabad, Gujarat 380009, India.; 2 Department of Pharmacology, L. M. College of Pharmacy, Niangua, Ahmedabad, Gujarat 380009, India.; 3 PharmD Section, L.M. College of Pharmacy, Ahmedabad, Gujarat 380009, India.; 4 Pharmacy Section, L.M. College of Pharmacy, Ahmedabad, Gujarat 380009, India.; 5 School of Pharmacy, Queen's University Belfast, 97 Lilburn Road, BT9 7BL, U.K

**Keywords:** nanoparticles, cancer, theranostics, photosensitive agents, photodynamic therapy, photothermal therapy

## Abstract

Cancer diagnosis and management have been a slow-evolving area in medical science. Conventional therapies have by far proved to have various limitations. Also, the concept of immunotherapy which was thought to revolutionize the management of cancer has presented its range of drawbacks. To overcome these limitations nanoparticulate-derived diagnostic and therapeutic strategies are emerging. These nanomaterials are to be explored as they serve as a prospect for cancer theranostics. Nanoparticles have a significant yet unclear role in screening as well as therapy of cancer. However, nanogels and Photodynamic therapy is one such approach to be developed in cancer theranostics. Photoactive cancer theranostics is a vivid area that might prove to help manage cancer. Also, the utilization of the quantum dots as a diagnostic tool and to selectively kill cancer cells, especially in CNS tumors. Additionally, the redox-sensitive micelles targeting the tumor microenvironment of the cancer are also an important theranostic tool. This review focuses on exploring various agents that are currently being studied or can further be studied as cancer theranostics.

## Introduction

According to recent data, there are over 1.9 million new cancer patients diagnosed in the year 2022. Nonetheless, 609360 deaths are attributed to cancer just in the united states and the number of cases is estimated to be increased by 12.5% by the end of the year 2025 1. Cancer is one of the most common causes of mortality and to be specific lung cancer and breast cancer is most common in men and women respectively 2. With advancements in the field of medicine, the management of cancer has evolved and progressed, but still, there are lots of problems associated with chemotherapy (CT) and radiation therapy (RT) regarding its effectiveness and improving the prognosis of the patient 3. In such a situation nanomedicine makes a good case that can be utilized to solve the issues with conventional CT related to drug delivery, distribution, and compatibility. And with advancements in nanomedicine approaches like theranostics can be utilized to diagnose and therapeutically treat the tumor with high accuracy 4, 5. Thus, the use of nanoparticles in the diagnosis and treatment of cancer is clubbed under the term “Nanotheranostics”.

The word theranostics comprises image-guided therapy which also consists of therapeutic potential and nanotheranostics includes the use of nanocarriers as a platform to deliver both diagnostic imaging modalities and therapeutic drugs 6. Nanotheranostics stretches more than nanomedicines which just include enhanced permeability and retention (EPR) effect 7. Whereas future medicine should not just focus on EPR effects on the tumor but should consider the tumor heterogeneity to achieve a better prognosis 8. And nanotheranostics can play a pivotal role in the stratification of the patient on basis of the therapeutic efficacy of the nanomedicine 9. With the help of nanotheranostics, the tumor microenvironment can be modulated artificially which results in optimum drug targeting 10. In addition to the EPR effect, it is also reducing opsonization which enhances the distribution and prevents the degradation of the medicine by the host's body. As a result, it reduces systemic toxicity and improves patient tolerance 11.

Early and precise diagnosis plays a pivotal role in determining the outcome of the treatment and for these various biomarkers and bio-imaging modalities are utilized 8, 12, 13. Certain organic, inorganic and hybrid based nanoparticulate materials are developed that can aid in diagnostic as well as therapeutic outcomes. These include gold-based nanoparticles, iron oxide nanoparticles, porphyrins, and many more theranostic agents that are mentioned below. It also provides opportunities to cross as many biological barriers and to interact with tumors more closely 14. This review summarizes the diagnostic as well as therapeutic applications of various theranostic agents along with its clinical applications.

## Diagnostic applications of nanotheranostics

The rationale of combining therapeutic and diagnostic purposes into a single agent using nanoparticles is emerging in current medicine. The capability of nanoparticles to penetrate deep into the tumor to deliver the therapeutic agent or the diagnostic agent raises the expectations from nanoparticulate agents to serve as a near-to-ideal theranostic agent 15. For diagnostics, non-invasive techniques such as computed tomography (CT), magnetic resonance (MRI), optical, ultrasound (US), and positron imaging tomography (PET) imaging can be utilized. Whereas for therapeutic purposes nanoparticulate agents can be used for gene silencing, delivery of antitumor agents, tumor targeting, and phototherapy.

### X-ray CT imaging

**Figure 1:**
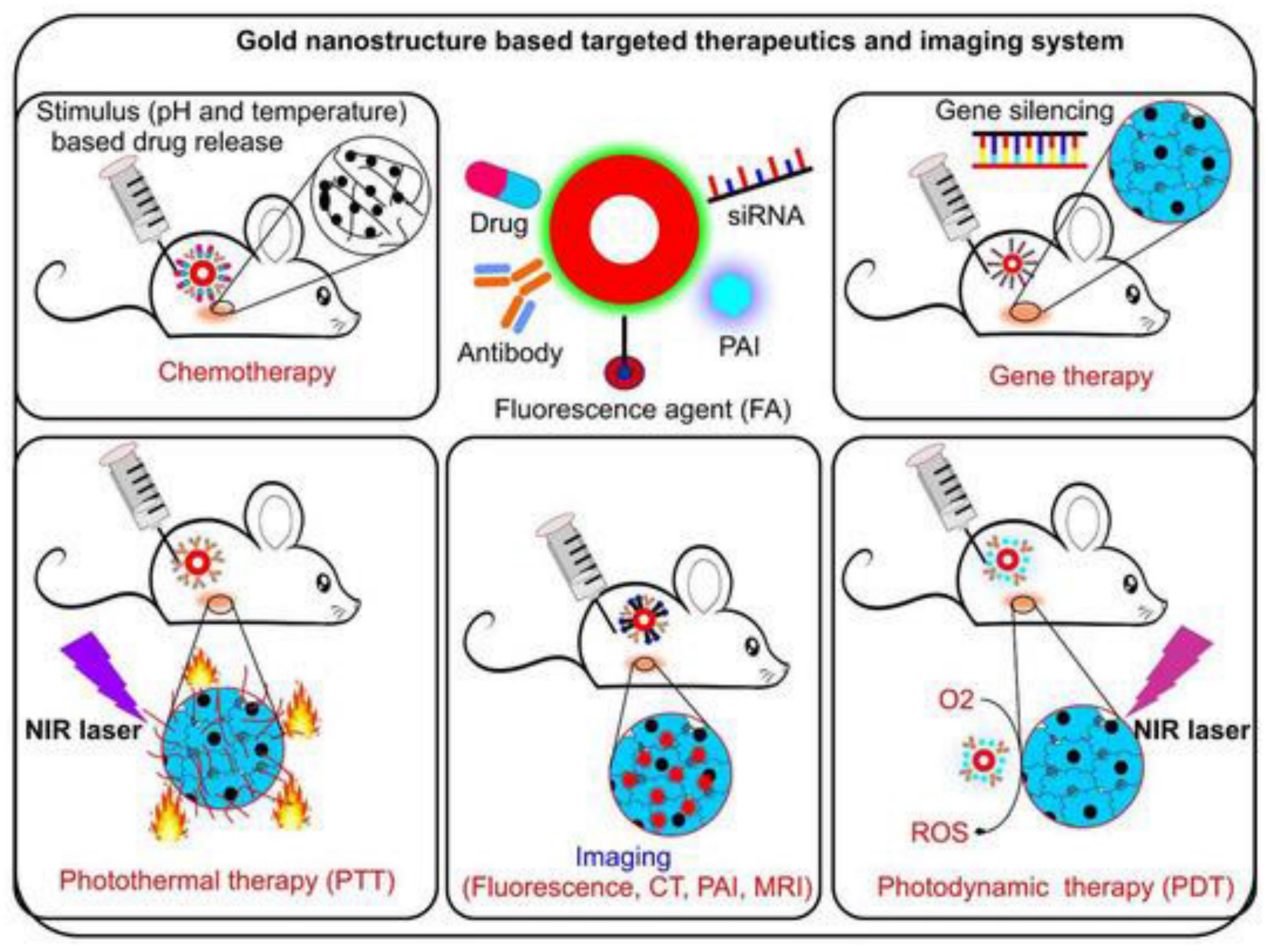
Gold nanoparticle is conjugated with various elements such as drug, antibody, siRNA and many more to form a stable conjugate. It enhances the photothermal therapy action, stimulates the drug release, enhance gene slicing and photodynamic therapy efficacy. (Adopted under CC BY 4 from) 23

CT imaging is a 3-dimensional extension of conventional X-ray that gives a clearer view by staking the cross-sectional images. The tissues are not naturally in contrast to each other thus a contrast agent is to be used. It is quite useful as it has deeper tissue penetration and high-resolution imaging 16. Usually, agents such as iodine, barium, or tungsten are used as contrast agents for CT imaging. Nanoparticulate contrast agents used include nano‐scale metal-organic frameworks (NMOFs), gold nanoparticles (AuNPs), and gold nanorods (GNRs). These have an upper hand over conventional contrast agents because the doses to be used can be significantly reduced by using nano-contrast agents. Also, renal toxicity significantly reduces compared to conventional contrast agents. AuNPs can ideally be used as X-ray contrast media owing to their higher molecular weight and lower risk of toxicities 17. Also, AuNPs are stable and easy to synthesize. Apart from its convenient use, various studies suggest the benefits of using AuNPs as nano-contrast agents. In a particular study carried out to check the sensitivity of AuNPs on hepatocellular carcinoma (HCC), it was found that ultrasmall AuNPs are much more sensitive for the diagnosis of HCC 18, 19. Also, a study was conducted where these nanoparticles were utilized for simultaneous tumor targeting of chemotherapeutic agents in prostate cancer 20. Thus, apart from being a useful contrast agent, these AuNPs can simultaneously be useful in targeting the chemotherapeutic agents at tumor sites. Au nanoparticles are hyperthermic and can destroy the tumor by the production of heat 21. Do refer Figure 1 for the application of Anup in therapeutics as well as diagnosis of cancer. They also improve the sensitivity RT and thus can be used as radiosensitizers. GNR is another example that can be used by coating it with different substances such as silica, cetyltrimethylammonium bromide, and so on 22.

### Magnetic resonance imaging

MRI utilizes radio waves and magnetic fields in order to serve as a non-invasive imaging technique. It gives cross sectional images and is widely useful for imaging of soft tissues. But due to its low sensitivity and longer time to obtain the images, contrast media are to be used to enhance its signals. These contrast agents can either be ferromagnetic or paramagnetic. Superparamagnetic iron oxide nanoparticles (SPIONs) as well as gadolinium are majorly used as a contrast agent in MRI 24, 25. Gadolinium is a T1 type of contrast agent that enhances the fatty tissue-based signals whereas SPIONs are T2 type of contrast agent that enhances water-based signals. Gadolinium when incorporated into PEG-coated liposomes were studied and found to have high applicability in imaging for neutral cell adhesion molecule 26 positive Kaposi's sarcoma 27. Other than these, genetically modified viral particles can also be utilized as a delivery system for nano theranostic agents including these contrast agents. A group of researchers utilized tobacco mosaic viral (TMV) nanoparticles to deliver gadolinium which led to increased relativity 28. SPIONs is a contrast agent of great importance owing to their biological inertness. Another major property of being highly tumor tissue specific. This is because it generates heat under magnetic field thus, maintaining least collateral damage to the healthy tissues 29. A significant reduction in tumor growth was observed along with almost nil liver or kidney toxicity when an alternating magnetic current was applied and heated the tumor up to 6°C within 20 minutes. For its theranostic application various chemotherapeutic agents can be loaded on to SPIONs. Doxorubicin along with SPIONs was developed for MRI imaging as well as CT. This loading was carried out by thermal crosslinking. Larger amount of doxorubicin was seen due to selective accumulation of nanoparticles 30. Apart from the basic chemotherapeutic agents, SPIONs are also useful along with gene-based cancer therapy. Gene therapy was combined with MRI in a study where a SPION-based shRNA system was developed 31. Polymeric nanoparticles combined with MRI contrast agents have shown great benefits in tumor targeted imaging. Various copolymers such as PLA-TPGS can be utilized for the same by combining it with MRI as well as fluorescent contrast agents making it a multimodal imaging approach. A contrast dye Cy5.5 when conjugated with iron nanoparticles, made possible 48-hour MRI monitoring 32. Apart from these, dendrimers are another delivery agent that are usually highly branched spherical polymers. A study showed a dendrimer designed for theranostic delivery that carried an MRI contrast diagnostic Cy5.5 along with paclitaxel. High cellular uptake along with reduced adverse effect on the non-target organs was seen on account of the dendrimer 33. But these dendrimers due to their positive surface charges have an inherited toxicity. Also, they are a little less stable as they tend to release the molecules rapidly sometimes before reaching the target sites.

### Optical imaging

Optical imaging is another non-invasive method of diagnosis that involves visualization of tissues via near infrared (IR), fluorescent as well as bioluminescent techniques. The dyes for the same are to be introduced into the patient before imaging 34. The delivery of these dyes, chemical methods such as conjugation with lipids or polymers or physical methods such as nano-encapsulation are important strategies for nanotheranostics. Conjugation of pyrene polymer with the dye for optical imaging into a nano-particulate formulation was proved to provide a high degree of tissue penetration and higher fluorescence intensity 35. Combining administration of various chemotherapeutic agents can be achieved along with the dyes after which optical imaging would be able to achieve not only tissue imaging but also drug distribution monitoring. Tumor targeted CT delivery can also be achieved by utilizing conjugation of nanoparticles with optical dyes. A fluorescent dye, Cy5.5 when conjugated with nanoparticulate glycol-chitosan and administered along with paclitaxel or doxorubicin showed much higher tumor specific actions thus, preventing its toxic effects on normal cells 36. Fibrin targeting is useful for brain tumors. In a murine model of glioma, a dye named Cy7 was administered for visualization of the tumor along with nanoparticulated conjugates showed significantly higher accumulation in the tumor tissues 37. Photosensitizing agents if used as a fluorescent dye pose dual functions of imaging as well as their role in photodynamic therapy (PDT). Various in vivo studies have proved the dual role of a photosensitive dye Ce6 in murine models. Apart from this, nanoparticulate based optical imaging dye delivery can also help in visualization of the drug as well as measuring of therapeutic efficacy of a chemotherapeutic drug 38. Thus, a nanoparticulate approach can bring out a lot of theranostic applications of these dyes via optical imaging. The bioavailability of fluorescent dyes was significantly improved on combining it with polymeric or magnetic nanoparticles such as PLA-TPGS or iron oxides. Fluorescent analysis in an ex vivo model showed a significant improvement in fluorescent intensity at various tumor sites on using the above-mentioned approach. The combination was also capable of crossing the blood brain barrier 39. Liposomes are bilayer amphiphilic agents that are important for delivery of theranostic agents 40. Fluorescent agents are usually loaded in the central aqueous compartment of the liposomes 41. A study was carried out where quantum dots were loaded into the liposomes and apomorphine as a therapeutic agent. Due to the small diameter of the liposomes, brain uptake of the quantum dots was significantly improved by 2.4-fold. Also, free quantum dots were rapidly eliminated from the brain tissues but liposome associated quantum dots gave a bright fluorescence up to 1 hour 42. Micelle formulation is another amphipathic formulation used for delivery of theranostic agents. TPGS micelles were prepared by a group of researchers containing supramagnetic iron oxides for diagnosis and therapy that showed improved magnetic properties with a high invitro cellular uptake in breast cancer cell lines 43.

### PET imaging

PET imaging provides noninvasive, highly sensitive 3D imaging of body tissues. It is usually combined with CT or MRI as PET alone lacks anatomical selectivity. PET contrast agents when delivered as nanoparticulate delivery can be very useful for tissue specific imaging, delivery of drugs as well as tumor targeting 44. A pH-dependent tumor targeting was carried out in an in vivo study. Here doxorubicin was conjugated with nanoparticles via hydrazone bonds and stayed intact at a normal physiological pH thus preventing the exposure and subsequent toxicity of the drug in normal tissues. It was released only in an acidic pH thus achieving tumor specific drug release. SPIONs used for PET imaging when coated with various nanoparticulated ligands and Ga (68) or Cu (64) were found to be useful for imaging as well as other therapeutic uses mentioned above 45. A concept of polymer-drug complex has been utilized for PET imaging by combining imaging contrast agents with an appropriate polymeric compound. For instance, N-(2-hydroxypropyl) methacrylamide (HPMA) copolymerized with gadolinium or Cu (64). When injected into tumor bearing mice, the radioactivity was found to be much higher in mice administered with the drug-polymer conjugate 46.

### Ultrasound imaging

As US imaging does not use any ionizing radiation or radio labelling, it is comparatively safer, cheaper and most widely used imaging technique. Various contrast agents that can be utilized for US imaging include microbubble and non-microbubble type agents. Conventional US imaging contrast agents have lower intrinsic specificity for malignant tissues 47. Microbubble contrast agents have high specificity and sensitivity for tumor cells. Thus, microbubbles are most used contrast agents to be used in combination with them. Microbubbles are made up of a gas filled core surrounded by a polymeric shell. The issue with this is the submicron sized bubbles are not capable of generating sufficient echogenic signals. Thus, in situ gas generating systems are developed. Anticancer drugs can be delivered using the same 48. A study was conducted that delivered doxorubicin by loading it with calcium carbonate nanoparticles with an organic polymeric backbone. This, when administered releases in situ CO2 in acidic solution. It was proved that there was a significant increase in the release rate of doxorubicin at acidic pH (tumor site) compared to neutral pH (healthy tissues) 49. Also, when combined it with PEG-poly(D-lactide) nanoparticles, the microbubble-based contrast agent was able to overcome the issue of lower aqueous solubility of paclitaxel 50. Various non microbubble modalities include echogenic liposomes, perfluorocarbon nanodroplets, and solid nanoparticles.

## Therapeutic applications of nanotheranostics

The applications of nanoparticulate derived agents in imaging and diagnostics have been described in the previous topic. Some of these agents can also be utilized for therapeutic purposes. An ideal theranostic agent should possess the intended pharmacological activity, stay into the body for a longer time. The therapeutic application that can be explored for cancer therapy using nanotheranostic agents can be majorly categorized into PDT, photothermal therapy (PTT) and CT. A working mechanism of these therapeutic modalities has been provided in figure 1. The agents used for the same can be organic, inorganic or hybrid in nature 51.

### Photodynamic therapy

PDT works on the principle that when certain tumor targeted agents are administered, they accumulate into the tumor tissue and release reactive oxygen species upon receiving external photo stimulation of a particular wavelength. The selection of photoactive agents to be utilized has to be very specific as they should possess specific tumor targeting and release of ROS to permanently damage tumor cells 52. Nanoparticles are introduced into this technology to overcome the issue of photobleaching 21. Most used agents for PDT include agents belonging to the tetrapyrrole family such as phthalocyanines and porphyrins as well as other dyes such as phenothiazines, phenalenones, squaraines, and indocyanine green 53. Various porphyrin based photosensitive agents that are used as nanotheranostics for PDT includes; protoporphyrin, 5,10,15,20-tetro (4-pyridyl) porphyrin, and hematoporphyrin. Other inorganic nanotheranostics that can be used for PDT include graphene quantum dots 54. Also, several hybrid agents such as Gd3+-Ce6-conjugated PEG-grafted poly-(maleic anhydride-alt-1-octadecene), Gd3+-Ce6, GdOF: Ln@SiO2, etc. The major advantage of using hybrid nanotheranostic agents is that the integration of PDT and PTT can be carried out to bring a synergistic effect of both modalities 55. As mentioned above dendrimers are a delivery system of various theranostic agents. A study was carried out where a novel dendrimer-based delivery platform was developed. A phthalocyanine derivative was prepared and entrapped into the dendrimer based nanocarrier system for delivering multimodal imaging agents and improving the efficacy of PDT. An increased cytotoxicity with a PDT effect up to 24 hours along with efficient internalization of imaging agents into tumor tissues was achieved 56.

### Photothermal therapy

**Figure 2:**
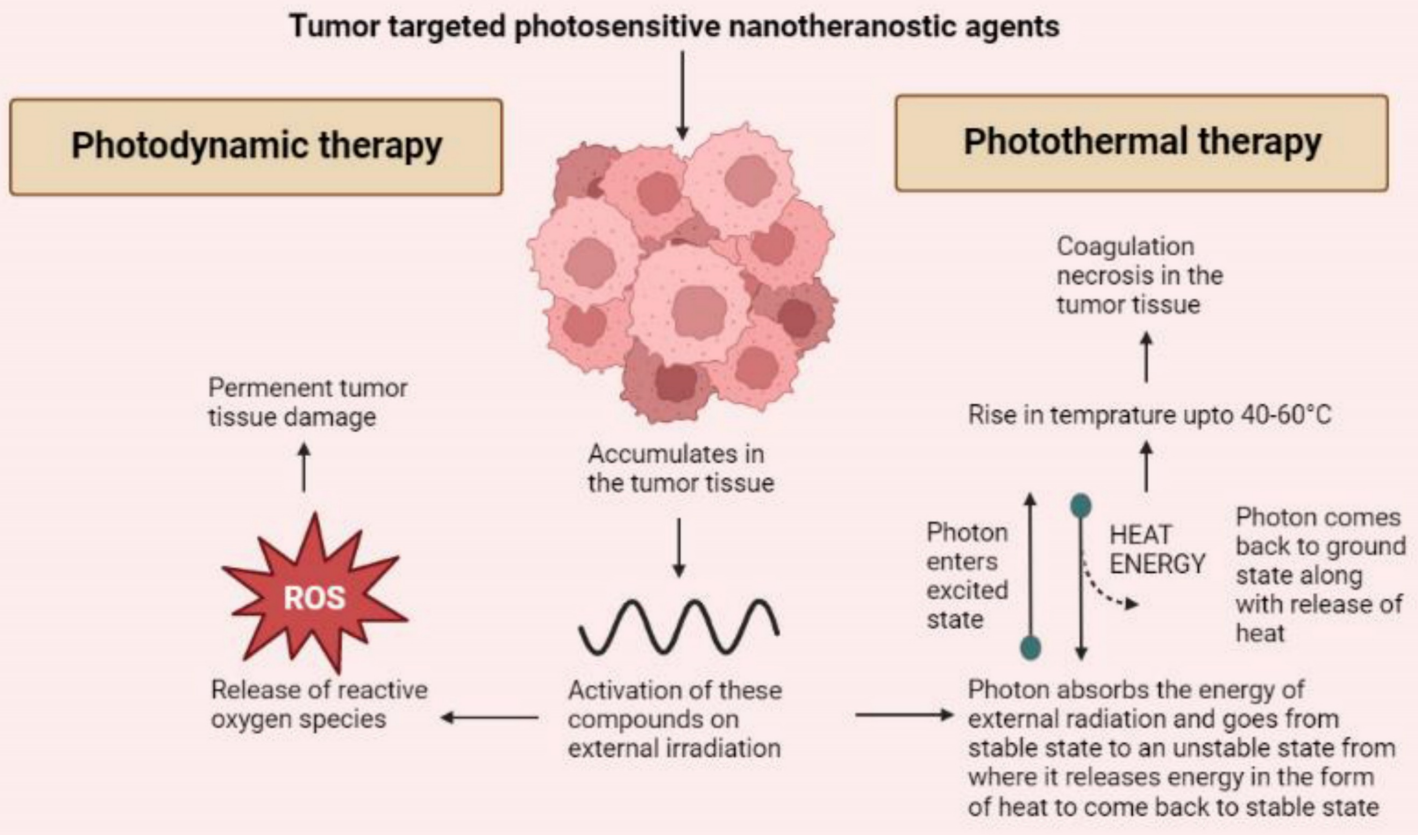
A basic mechanism through which the anti-tumor effects of photodynamic and photothermal therapies are mediated.

The technology behind PTT is using such contrast or nanotheranostic agents that can convert photo energy to thermal energy and thus, bring about tumor cellular necrosis by inducing hyperthermia 57. Usually, near IR range is used to initiate the release of heat by activating these photosensitive agents 58. On exposure of these photosensitive agents to external irradiation, the electrons of the photosensitive agents absorb a photon and get excited moving to an unstable state. When returning to ground state, the release of energy takes place via heat 59. PTT targets tumor tissues by using agents that specifically accumulate in the tumor cells thus sparing the healthy cells. The irradiation to the photosensitive agents leads to a rise in temperature up to 40 to 60°C leading to coagulation necrosis 60. Like PDT, various organic, inorganic and hybrid agents can be utilized for PTT. Majorly inorganic agents are used for PTT including metallic, carbon nanostructures, and quantum dots based on their optical and thermal properties. Inorganic agents such as gold, silver, iron oxide, graphene, carbon nanotubes are being used. But these inorganic agents pose an issue of bioavailability and toxicity. Thus, use of hybrid nanoparticulate agents is more widely preferred 61. Organic agents are also used as theranostics for PTT. These include porphyrin-based agents such as pyropheophorbide, and purpurin whereas cyanine-based agents include CySCOOH, cypate, and DiR. Other organic agents such as polyaniline, gallic acid, and Prussian blue are also used as light triggered nanotheranostics agents for PTT 54. Hybrid nanotheranostics are preferred as they help in overcoming the shortcoming of other agents by having a good distribution and elimination profile and being relatively less toxic. Apart from this, the hybrid agents also help in achieving synergy of PDT and PTT as mentioned above. This synergism not only leads to increased tumor cell death but also helps in improving the sensitivity of the tumor to other treatment modalities such as CT 62. Magnetic nanoparticles such as iron oxide or certain carbohydrates when coated on the surface of biopolymers lead to induction in the hyperthermia effect of PTT. Dendrimers made of polypropylenimine were known to destroy ovarian tumor cells using a synergistic effect of PDT and PTT 63. Liposome or lipid-based nanoparticles such as PEG‐PLGA nanoparticles were used to encapsulate ICG dye and a TLR7 agonist that produced anti-tumor antigens during PTT. It was successful in preventing metastasis in an orthotropic model. Another study used a nanohybrid agent CuS/Cu2S/Au in order to improve the PTT targeting and efficacy. It occurred because the improved efficacy of photothermal conversion provided larger cavities and a mesoporous shell 64 (Figure 2).

### Chemotherapy

Conventionally used chemotherapeutic agents suffer from the major disadvantage of being toxic to healthy tissues thus leading to severe adverse effects. This issue can be conveniently bypassed when the chemotherapeutic drugs are given in a tumor-targeted manner. For such tumor targeting, nanoparticles offer an important ability to make sure that no off targeted drug delivery takes place and transports the therapeutic agents to particular tumor sites only 65. Various nanocarriers can be conjugated with chemotherapeutic agents such as paclitaxel, doxorubicin, gemcitabine, carboplatin, etc. to be administered directly targeting the tumor tissues. Organic nanotheranostics agents such as benzoporphyrin derivatives, hematoporphyrin, indocyanine green, and Prussian blue are combined with chemotherapeutic drugs and administered. A hybrid nanoparticulate agent namely rGO@Au-NR NV i.e., reduced graphene oxide coated with gold nanoparticles is used to deliver certain chemotherapeutic drugs such as doxorubicin 66. Gold nanoparticles when administered with paclitaxel, can be utilized for tumor-targeted therapy as well as imaging to track the tumor size 67. Au nanotheranostics have proved to be of great importance in targeting ovarian and breast tumors when combined with gemcitabine or doxorubicin 68, 69. Previous studies have conclusively proven that nanoparticulate drug has more efficacy than conventional molecule. One such study compared polymeric doxorubicin from a nanoparticle generator to doxorubicin alone in metastatic breast cancer where the former had significantly improved efficacy compared to the later 70. Also, doxorubicin along with silica nanoparticles and graphene dots can be used for tumor-targeted therapy as well as fluorescent imaging 71. Apart from this, technologies such as dendrimers, liposomes, polymeric nanoparticle micelles, and solid lipid nanoparticles can be utilized for the delivery of chemotherapeutic drugs 72. Other inorganic-based nanotheranostics include MoS2 nanosheets, single walled carbon nanotubes, and tungsten sulfide 54. Another study utilized poly(allylamine)‐citraconic anhydride/doxorubicin (PAH‐Cit/DOX) nanoparticles to deliver doxorubicin nanoparticles to the tumor site. The nanoparticles dissociate from the carrier system at a lower pH in presence in endosomes or lysosomes. Subcellular analysis was carried out in order to track the doxorubicin release in different compartments using fluorescence lifetime imaging microscopy (FLIM) technique. It showed significant difference between release rates of doxorubicin in different compartments 73. An ester poly(β‐amino) core and hyaluronic acid shell were utilized as carriers for doxorubicin. It was concluded that hyaluronic acid was useful in improving the transmission of doxorubicin thus allowing to overcome its resistance in breast cancer 74. Another shell material that was evaluated was PLGA‐alginate core‐shell particles. But it actually made the drug release slower and acted as a barrier in drug diffusion 75. The drug-polymer complex is also an interesting theranostic application where various polymers can be combined with drug molecules to obtain better targeting. N-(2-hydroxypropyl) methacrylamide (HPMA)-doxorubicin is one such drug-polymer complex that is under phase 1 clinical evaluation 76. Apart from that, polymeric nanoparticles such as Poly(D, L-lactide-co-glycolide) (PLGA), PLA-TPGS and poly(D,L-lactide-co-glycolide) -polyethylene glycol (PLGA-PEG) and many more can be used for co-delivering therapeutic as well as diagnostic agents. These have high prospective value as they have a good storage stability along with being least toxic inside the body 77. A preclinical study had shown a 5.7-to-8-fold improvement in drug delivery of docetaxel that was co-formulated with PLGA nanoparticles. These polymeric nanoparticles are highly targeting because they enter the cells via endocytosis and then internalized into endosomes where they can be degraded 78. An invitro study co-formulated docetaxel with PLA-TPGS based polymer which showed much specific folate cell targeting in breast cancer cell lines 79. Solid lipid nanoparticles are basically biodegradable lipid nanoparticles such as triglycerides. These serve as a good platform for delivering of therapeutic agents as owning to their small size, they can penetrate the blood brain barrier. They can be delivered directly into the lymphatic system for improving bioavailability 80. A study was carried out that combined the drug paclitaxel and Bcl2 targeting siRNA along with quantum dots loaded solid lipid nanoparticles. It was designed in a way where paclitaxel and quantum dots were loaded into the inner lipid shell whereas the anionic siRNA into the outer shell of the solid lipid nanoparticles. A stronger synergistic anticancer effect was seen on the lung carcinoma cells 81. Small bilayer vesicles with an internal aqueous layer and an outer lipid coat known as liposomes have been widely utilized as carriers for theranostic agents. Mostly the chemotherapeutic drugs are formulated into the outer coat of the liposomes 82. TPGS-coated liposomes were loaded with docetaxel and exposed to in vitro brain tumor cell lines where they showed significantly higher efficacy 83. In another such study, a higher cellular uptake was noted when folic acid was used to target folate receptor overexpressing breast cancer cell lines. Loading of liposomes with quantum dots for fluorescent imaging and camptothecin, irinotecan for antitumor effects showed bright fluorescence lasting for up to 24 hours and higher accumulation of the drugs in solid tumors 84. Miscellas are another type of colloidal delivery system with a hydrophobic core for drug or diagnostic loading and a hydrophilic coat for targeted agent 85. Genexol-PM is one such example of a micellar formulation loaded with paclitaxel which is currently being evaluated in phase 1 clinical trials 86. Miscellas of hyaluronic acid-doxorubicin were assembled which proved to be successful as theranostic platforms or delivery systems 87.

## Clinical use of nanotheranostics

In addition to the diagnostic and therapeutic functions of nanotheranostics platforms, there are a range of other applications. This application ranges from patient risk identification and stratification of the sub-population, monitoring the intra-tumor drug distribution, a response to CT, and imaging-guided therapies 88. These applications play an important role, especially in the case of tumor heterogeneity where personalized therapy is given.

### Patient risk identification/stratification of sub-population

Generally, the nanomedicines are administered to the patient in different dimensions to understand the optimal drug uptake and utilization by the body. But it is atypical for a scientist to administer the same nanomedicine to two subjects having the same tumor characteristics to subgroup based on the drug uptake by the tumor 89. This brings a halt to the belief that xenogeneic model of tumors share similar characteristics and generate a similar response to the drug administered. An experiment was done to provide evidence to this hypothesis, in which the xenogeneic model of breast tumor were prepared and radiolabelled iodine liposome was administered and subjects were grouped into 2 groups good prognosis and bad prognosis based on their iodine uptake. Furthermore, both groups were treated with CT and showed better results in the good prognosis group compared to the subjects in another group. Thus, it can be understood that even in the xenogeneic model the tumor variation has a defining impact in determining the outcome of the therapy. These variations in the tumor characteristics are found to be more significant in larger animals 90. Recently, ferumoxytol-based MRI was used to identify the effectiveness of paclitaxel-based nanomedicine. Ferumoxytol was initially used to treat iron deficiency in patients with chronic kidney disease. There is a need to develop radio-labelled liposomes (loaded with drugs) to serve as nanotheranostics formulations 91. As of now, there is no nanotheranostics formulation available for approval much research is to be done to translate the idea into clinical practice 90.

### Monitoring intra-tumor drug distribution

The distribution of the drug intratumorally is an important criterion in defining the therapeutic effectiveness of the drug. The drug distribution inside the tumor is determined by blood flow to the tumor, interstitial fluid pressure, and thickness of the tumor. For any drug, it is difficult to reach the central hypoxic portion of the tumor because of the poor blood supply. The central mass of the tumor is responsible for tumor heterogeneity and cancer stem cells are ought to be present in the center part 92. In preclinical laboratory settings, intravital microscopy is used to study the drug distribution of nanomedicines intratumorally for example it was used to study the drug distribution of temperature-sensitive liposomes of doxorubicin 93. The use of intravital microscopy is not possible in clinical settings hence MRI based techniques are used to study the interaction between intratumorally behaviours and nanomedicine 94.

Some nanotheranostics formulations are designed in such a way that once administered in the body they are activated by exogenous stimuli rather than endogenous stimuli such as X-rays, heat, and ultrasound. If we take an example of fibrosarcoma tumors in rate when temperature-sensitive liposome of doxorubicin is administered, better intratumor drug distribution was observed when administered while hyperthermia rather than afterward 95.

### Image-guided focus therapies

The image-guided focus therapies mainly comprise nanomedicines enabled via PDT and PTT; it had lesser chances of toxicity. In this method, efficacy is determined via PDT and PTT rather than intratumorally drug distribution is seen in chemotherapeutic agents 96, 97. The major limitation to image-guided focus therapies is it is used in tumors that are proximal to the skin as PDT and PTT lack penetration. The PTT agents which dissipate heat make them a good candidate for photo-acoustic imaging (PAI) 98. Thus, we can track the PTT agent with the help of PAI and save the resources for an extra imaging modality. A study done in a mouse model used PAI imaging, in which gold nanospheres were administered, and then the mouse was given photothermal ablation to improve the tumor selectivity 99.

### Response monitoring

Monitoring the response after administering the drug is a good idea to obtain the immediate prognosis of the patient post-therapy. It helps us in planning the further treatment plan of the patient. In laboratory settings, it is done by measuring the tumor dimensions using scales. However, these are not practically possible after every dosing schedule 100. So many researchers suggest the use of nanotheranostics to solve the issues. The tumor microenvironment (TME) changes with the response to the therapy as TME has reduced pH, hypoxia, and increased activity of matrix metalloproteinase activity during tumor progression. When therapy is showing a response, these abnormalities are tended to decrease and normal physiology is achieved which can be traced via the use of various techniques 101. The optical nanoprobes having a sharp pH response are used but because of limited tissue penetration of light, it is not yet been clinically used. As of now, it seems more practical to use separate imaging techniques to track the progress of nanomedicine 102.

### Targeted and improvised delivery

**Figure 3:**
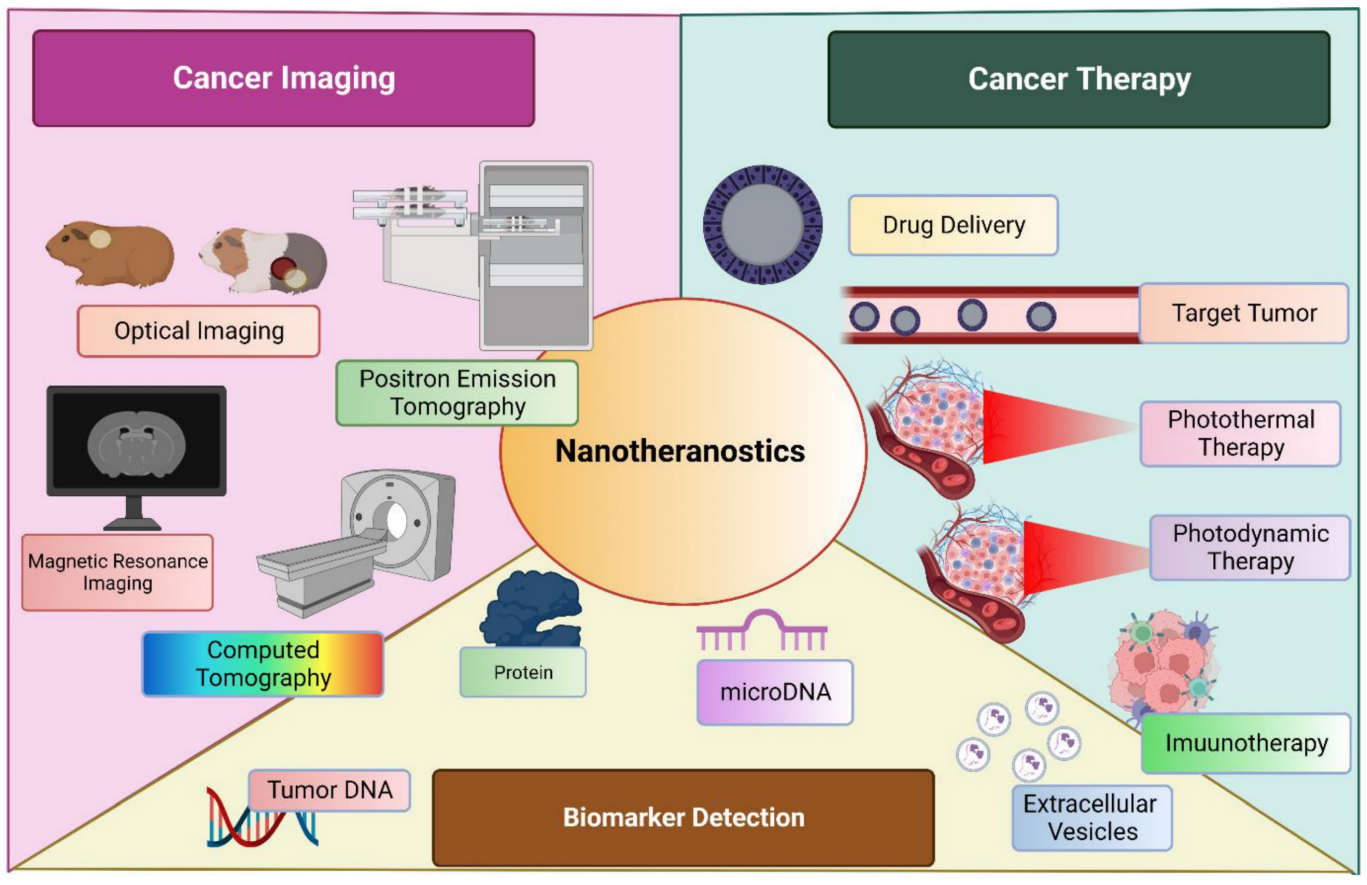
Nanotheranostics is employed broadly into three sectors; cancer imaging, cancer management and biomarker detection.

Nanomedicine has evolved itself in various fields to improve the targeted delivery, enhance the therapeutic action, filling the voids of current practice and many more. Cancer immunotherapy is an emerging filed which incorporate the mRNA vaccine as an promising method 103. Although, holding a great potential, is drawn back by its limitation such as innate immunogenicity, ineffective in in-vivo delivery and instability. Various formulation tactics such as addition of lipid nanoparticle (LNPs), peptides and many more. USFDA approved LNP-loaded mRNA for management of COVID-19 by boosting the immunity. 104, 105 Another widely accepted application of nanoparticles was observed in delivery as metalloporphyrin. These molecules are encapsulated within the porphyrin nanoparticle without any specific need of chelator. These entities have wide range of application including therapeutics, imaging, analytical, and diagnosis. This chelated moiety has greater tendency to bioconjugate with other molecules as well to co-deliver them to a targeted site of action. 106 A diagrammatic representation of nanotheranostics, being employed for multiple use in management of cancer is described in Figure 3.

## Future challenges and problems

The major problem associated with nanomedicine is tumor heterogeneity and an overly simplified pharmacokinetic model of the tumor. And based on this it is believed that the concentration of the drug inside the tumor depends on just the drug available in the bloodstream for a longer period of time 107. This model didn't consider various factors such as vascular density, distribution of the blood vessel in the tumor, the thickness of stromal cells, and the presence of macrophages. Each of these factors plays a distinctive role in intertumoral drug distribution and each tumor should be treated as a separate organ 108, 109. In general, all tumors are considered to be highly vascular organs but there is a lot of variation in different types of tumors regarding vascularity 110. For example, squamous cell carcinoma has comparatively lesser vessels than colorectal carcinoma. This tumor vascular density can be assessed by various imaging techniques including MRI, CT, and so on. Other than tumor vasculature the leakiness of the tumor vessels also plays an important role. It is determined in the laboratory setting by the use of Evan's blue dye. In clinical settings, MRI can be used to determine leakiness. Recently a drug gadofosveset, an albumin binding drug was approved to determine the leakiness in tumors using contrast MRI 111. Thus, thorough identification of the tumor vasculature and leakiness aids in achieving better prognosis.

## Conclusion

From diagnosis to therapy, nanotheranostics can be of great aid in patient care. They can be utilized as tools for diagnosis and tumor monitoring, delivering chemotherapeutic agents, being useful as anti-tumor agents, and providing a synergistic effect with other anticancer modalities. Till now these agents have only been tried in different formulations by different technologies in *in-vitro* and preclinical *in vivo* studies. Even though a lot of research has been done in the field of nanotheranostics, there are not many clinical studies done explaining the effectiveness of nanotheranostics in patient care. For that, we need to first understand the role and characteristics of nanomedicine as a diagnostic and therapeutic tool. If that is achieved, it could revolutionize the work of tumor diagnosis and therapeutics.
